# Genetic analysis and molecular mapping of QTLs for resistance to rice black-streaked dwarf disease in rice

**DOI:** 10.1038/srep10509

**Published:** 2015-07-22

**Authors:** Tong Zhou, Linlin Du, Lijiao Wang, Ying Wang, Cunyi Gao, Ying Lan, Feng Sun, Yongjian Fan, Guoliang Wang, Yijun Zhou

**Affiliations:** 1 Institute of Plant Protection, Jiangsu Academy of Agricultural Sciences, Jiangsu Technical Service Center of Diagnosis and Detection for Plant Virus Diseases, Nanjing 210014, China; 2 Institute of Plant Protection, Chinese Academy of Agricultural Sciences, Beijing 100193, China; 3 Scientific Observation and Experimental Station of Crop Pests in Nanjing, Ministry of Agriculture, Nanjing 210014, China

## Abstract

Rice black-streaked dwarf disease, caused by *rice black-streaked dwarf virus* (RBSDV), is transmitted by small brown planthoppers (*Laodelphax striatellus* Fallén, SBPH) and causes severe yield loss in epidemic years in China and other East Asian countries. Breeding for resistance to RBSDV is a promising strategy to control the disease. We identified Tetep that showed resistance to RBSDV using a field test and artificial inoculation test. An evaluation of the resistance mechanism revealed that Tetep was resistant to RBSDV but not to SBPH. Genetic analysis showed that the resistance of Tetep to RBSDV was controlled by quantitative trait loci (QTLs). Three new QTLs for RBSDV resistance were identified in this study, i.e., *qRBSDV-3*, *qRBSDV-10* and *qRBSDV-11*. The LOD scores of *qRBSDV-3*, *qRBSDV-10* and *qRBSDV-11* were 4.07, 2.24 and 2.21, accounting for 17.5%, 0.3% and 12.4% of the total phenotypic variation, respectively. All the resistance loci identified in this study were associated with virus resistance genes. The alleles for enhancing resistance on chromosomes 3 and 11 originated from Tetep, whereas the other allele on chromosome 10 originated from a susceptible parent. The identified new resistance QTLs in this study are useful resources for efficiently breeding resistant rice cultivars to RBSDV.

Rice black-streaked dwarf disease is caused by the rice black-streaked dwarf virus (RBSDV), which is transmitted by small brown planthoppers (*Laodelphax striatellus* Fallén, SBPH) in a persistent manner but is not transmitted to offspring through the ovary^1^,^2^,^3^,^4^. Rice plants infected with RBSDV exhibit typical symptoms, such as stunting, dark-green leaves, and white waxy or black-streaked swollen areas along the veins on the stem and abaxial leaf surface^1^,^4^,^5^,^6^. This disease leads to severe rice yield losses in China and other East Asian countries^7^.

The identification of resistance genes and the development of resistant cultivars are economical and effective strategies for controlling this viral disease and have proven useful for controlling rice stripe disease^8^,^9^,^10^, which shares the same transmission vector as RBSDV and caused severe losses in rice fields in China at the start of this century. To date, most of the varieties found to be resistant to rice black-streaked dwarf disease have inherited the trait in a quantitative manner, and several quantitative trait loci (QTLs) have been identified^11^,^12^,^13^. Because the mechanism of resistance to rice black-streaked dwarf disease can be divided into three types, i.e., resistance against the virus, resistance against the vector or resistance against both the virus and the vector, resistant cultivars should be implemented differently within different contexts. Although plant breeders and geneticists have devoted increasing attention to understand resistance mechanisms to rival diseases, the mechanisms underlying the resistance have not yet been revealed to date.

In this study, we attempted to understand the mechanism and inheritance mode of resistance to rice black-streaked dwarf disease in the resistant cultivar Tetep. We performed a QTL analysis to map the resistance to the virus by generating a genetic linkage map and collecting resistance phenotypes in the progenies of a genetic cross between Tetep and the susceptible cultivar Huaidao No.5. Our data have provided useful information that can enhance our understanding of the molecular mechanism underlying RBSDV resistance in Tetep and assist in the rapid development of rice cultivars conferring resistance to RBSDV.

## Results

### Evaluation of resistance to RBSDV

An antibiosis test showed that the average survival rates of SBPH on Wuyujing No. 3, Huaidao No. 5 and Tetep were higher than the survival rate on IR50, with a highly significant difference (P < 0.01) (Table 1). These data indicate that Wuyujing No. 3, Huaidao No. 5 and Tetep did not exhibit antibiosis. In the non-preference test, the average number of SBPH on each single seedling varied among the four rice varieties, and there was a significant difference between IR50 and Huaidao No. 5. In contrast, no significant difference was observed between IR50 and Tetep (P < 0.05), which demonstrated that Tetep had a weak non-preference. Therefore, Tetep likely has weak resistance to SBPH.

The results of dot-ELISA revealed that the rates of infection of SBPH with RBSDV were 27% and 35% from plants grown in the experimental farms of the Institute of Jianhu Agricultural Sciences (JH) and the plant protective station of Guanyun (GY) in Jiangsu Province, respectively. Because the total numbers of SBPH were 7.9 × 10^6^ and 6.9 ×10^6^ per hm^2^ in 2010^14^, the effective inoculation amounts were 2.13 × 10^6^ and 2.42 × 10^6^ per hm^2^ in JH and GY, respectively. Based on 3 ×10^5^ plants per hm^2^, there were likely to be approximately 7 and 8 SBPH per plant in JH and GY, respectively.

**Figure 1:**
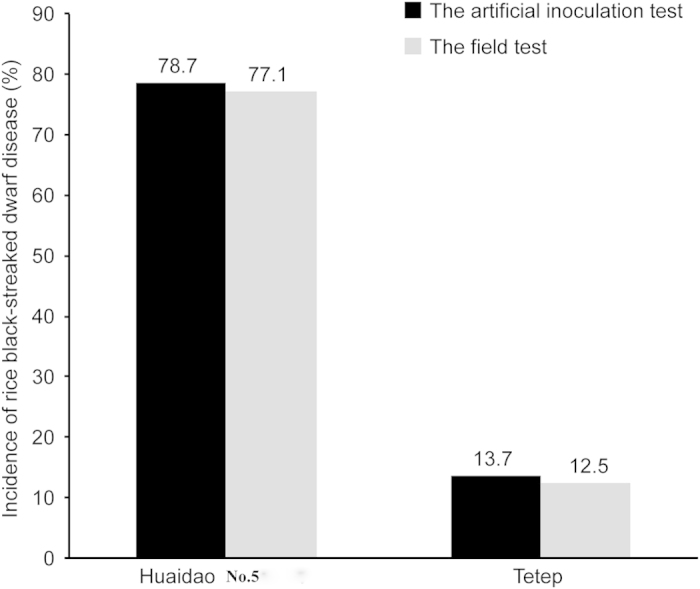
RBSDV incidences in Huaidao No.5 and Tetep as determined by the artificial inoculation and the field tests.

The incidences of disease on Huaidao No. 5 were 77.1% and 78.7% in the field test and artificial inoculation test, respectively (Fig. 1), which indicated the accuracy of the evaluation methods. The incidences of disease on Tetep were found to be 12.5% and 13.7% using the field and artificial inoculation test, respectively, these values are significantly lower than those for Huaidao No. 5. Additionally, there was no significant difference between the results of two methods, demonstrating the accuracy of the artificial inoculation test and also indicating that the resistance of Tetep is mainly due to resistance against RBSDV.

### Phenotypic analysis of the progeny of the cross between Tetep and Huaidao No. 5

**Figure 2:**
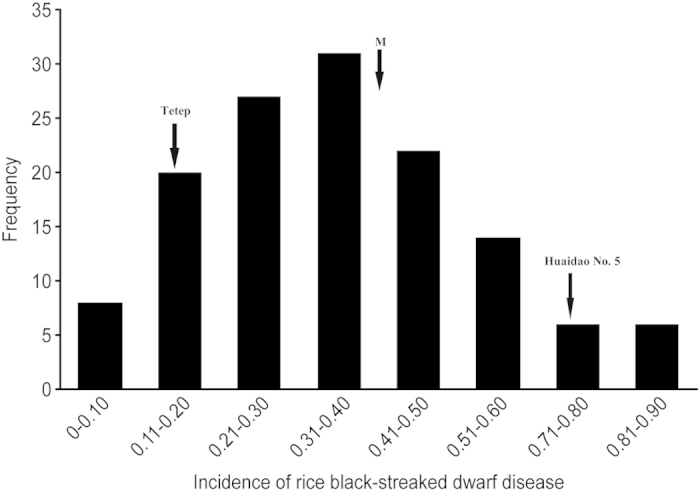
Frequency distribution of the F_2:3_ families with rice black-streaked dwarf disease as determined by artificial inoculation identification.

RBSDV resistance was evaluated for each individual in F_2:3_ families and for the two parents by the artificial inoculation test. The average incidence in each F_2:3_ family represents the incidence in its corresponding F_2_ individual (Fig. 2). The distribution of disease incidence was not bimodal and showed a continuous distribution from 1–100%, suggesting that the RBSDV resistance in Tetep is controlled by QTLs.

### Construction of a genetic linkage map and QTL analysis

Out of 842 markers examined, 160 SSR markers were found to be polymorphic between the two parents, for a ratio of 19.01%. A linkage map was constructed that included 12 linkage groups and spanned a total of 2179.6 cM of genetic distance, with an average of 17.16 cM among 127 SSR polymorphic markers. Because these SSR markers were evenly distributed among 12 chromosomes, the linkage map was suitable for QTL detection.

**Figure 3:**
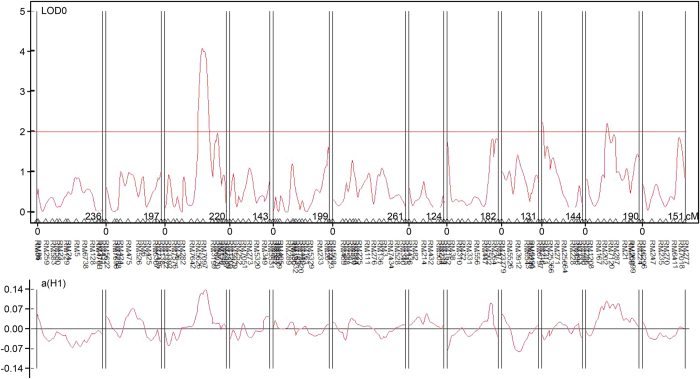
Analysis of QTLs for resistance to RBSDV.

**Figure 4:**
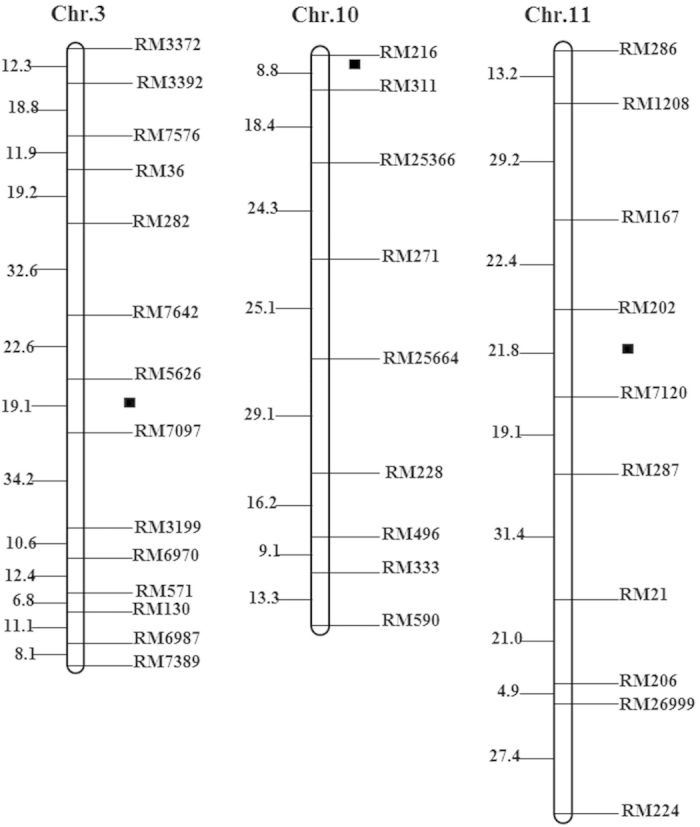
QTLs for resistance to RBSDV identified in F_2_ lines derived from Huaidao No.5/Tetep. ■ indicates a QTL for resistance to RBSDV.

QTL identification using the composite interval mapping (CIM) method revealed a total of three QTLs, i.e., *qRBSDV-3*, *qRBSDV-10* and *qRBSDV-11*, for resistance to RBSDV at the marker intervals of RM5626-RM7097, RM216-RM311 and RM202-RM7120 on chromosomes 3, 10 and 11, respectively (Table 2, Figs. 3, 4). The LOD values of *qRBSDV-3*, *qRBSDV-10* and *qRBSDV-11* were 4.07, 2.24 and 2.21, which accounted for 17.5%, 0.3% and 12.4% of the total phenotypic variation, respectively. In addition, *qRBSDV-3* and *qRBSDV-11* were derived from Tetep and had additive effects on the resistance to RBSDV.

## Discussion

The identification of genes conferring resistance to plant diseases and the development of resistant cultivars are considered the most economic, effective and environmentally friendly measure for controlling plant diseases^15^,^16^,^17^. Researchers have tried to identify the genetic factors that control the resistance to rice black-streaked dwarf disease for a long time. Some reports described the localization of genes conferring resistance to this disease^11^,^12^,^13^, and several “hot spot” regions on chromosomes 3 and 11 were identified. These results suggested that rice might have developed multiple mechanisms of resistance to rice black-streaked dwarf disease during the long-term co-evolution among the virus, the plant and the vector. Because a field test was the only method used for phenotypic analysis in the above reports, the resistance mechanisms of the identified genes have not yet been clarified, i.e., it is not known whether rice plants are resistant to SBPH, RBSDV or both. This lack of information has slowed the progress of breeding programs. In our previous study, we developed an artificial inoculation identification method for evaluating the RBSDV resistance level of rice varieties after overcoming three obstacles: interference from *southern rice black-streaked dwarf virus* (SRBSDV) due to similar symptoms, the preservation of the virus sources used for the artificial inoculation identification, and interference from *rice stripe virus* (RSV), which can be transmitted to offspring through the ovary^14^. The resistance loci identified in this study were verified as RBSDV resistance genes using the artificial inoculation identification, which was verified based on the results of the resistance analysis in Tetep.

**Figure 5:**
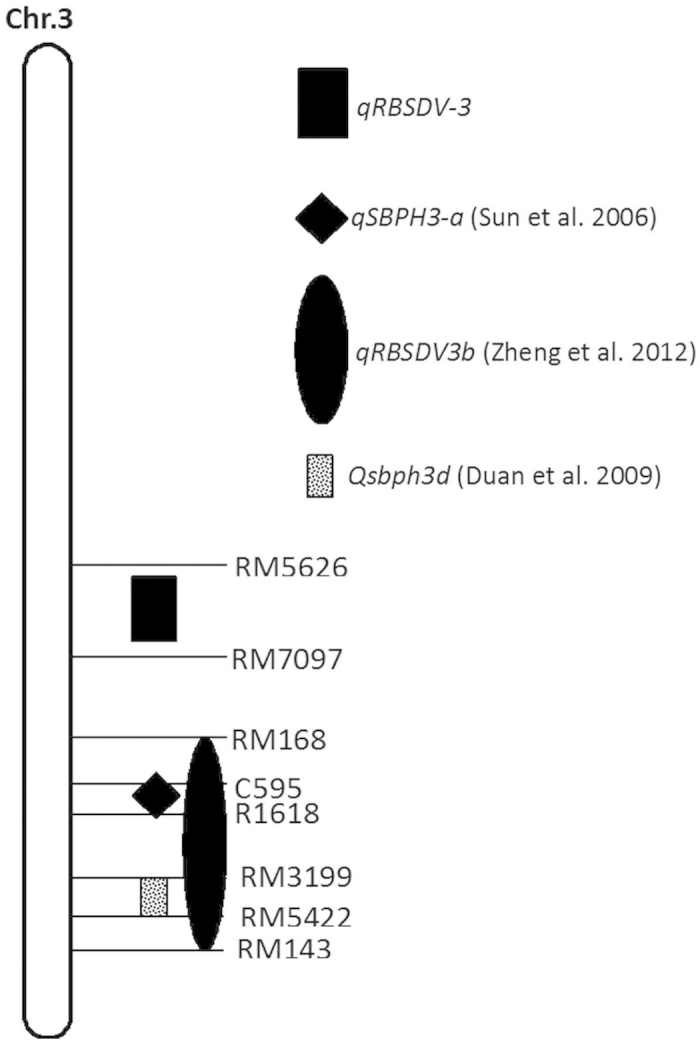
Comparison of the resistant loci around *qRBSDV-3.*

*qRBSDV-3*, which was identified in this study, is located in a hot spot on chromosome 3 that contains genes conferring resistance to RBSDV or SBPH, including *qRBSDV3*, *qRBSDV3a*, *qRBSDV3b*, *qSBPH3-a* and *Qsbph3d*^12^,^13^,^18^,^19^. By comparative mapping using common DNA markers, *qRBSDV-3* was found to be close to the region harboring *qRBSDV3b*, which also harbors *Qsbph3d* and *qSBPH3-a*. However, *qRBSDV-3* and *qRBSDV3b* are not the same locus, as they are mapped to different regions. In addition, Zheng *et al.*^13^ reported that *qRBSDV3b* is only involved in SBPH resistance. Additionally, *qRBSDV3a*^13^ and *qRBSDV3*^12^, which mapped to the same region, are quite distant from *qRBSDV-3* (Fig. 5). These results indicate that *qRBSDV-3* in our study is a new QTL that only confers resistance to RBSDV.

**Figure 6:**
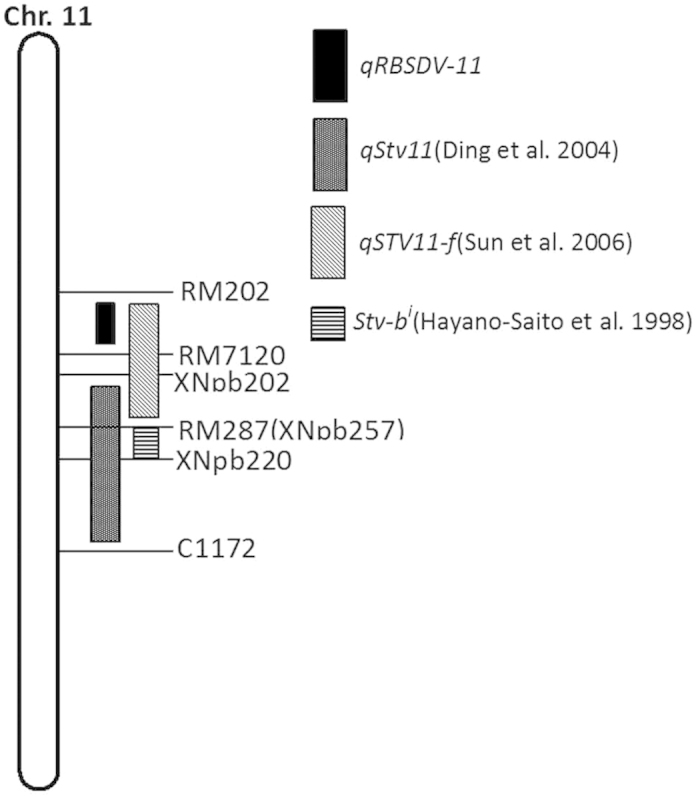
Comparison of the resistant loci around *qRBSDV-11.*

*qRBSDV-10* mapped to the short arm of chromosome 10 in our study. By comparative mapping using common DNA markers, portions of *qRBSDV-10* were found to overlap with the region of *qSTV10* that confers RSV resistance^20^. *qRBSDV-11* is located on the long arm of chromosome 11, however, *qRBSDV11*^13^ and another two loci reported to confer RBSDV resistance^11^ mapped to the short arm of chromosome 11. Thus, *qRBSDV-11* is a new QTL found in this study. To date, some major QTLs for rice stripe disease resistance have also been identified on chromosome 11, such as *qSTV11-f*, *stv-b*^*i*^ and *qStv11*^20^,^21^,^22^. According to the comparative mapping analysis, *qSTV11-f *20 and *qStv11*^22^ were localized to the same region as *qRBSDV-11* in this study (Fig. 6). The loci responsible for resistance to different types of pathogens or pests are thought to be clustered in specific chromosomal regions in many organisms^20^,^21^,^22^,^23^,^24^,^25^,^26^. Indeed, many reports have demonstrated that the region described above is a hot spot for genes that confer resistance to diseases, including rice blast, rice bacterial leaf blight and rice stripe disease, or pests, including *Nephotettix cincticeps, Nephotettix virescens* and SBPH. These data indicate that this region might play an important role in the co-evolution between plants and parasites, thus, more attention should be devoted to this region in resistance breeding programs.

Unlike regular pests, SBPH acts as a transmission vector and causes plant loss by spreading viruses other than directly attacking. Thus, the identification of RBSDV resistance genes becomes more urgent to reduce yield loss from SBPH attacks, which are currently occurring throughout China. In this regard, loci that only confer resistance to the virus, such as those discovered in this study, could be more effective and can be further improved by fine mapping to identify tightly linked markers. These markers can be used in marker-aided selection aimed at developing cultivars that are resistant to rice black-streaked dwarf disease.

## Materials and Methods

### Plant materials

Tetep, an *indica* variety from Vietnam, was used as the resistant parent, and Huaidao No.5, a *japonica* cultivar that is popular in Jiangsu province but highly susceptible to RBSDV, was selected as the male parent. All mapping populations were developed from the same cross between Huaidao No.5 and Tetep. In 2009, the F_1_ population was grown at the experimental station at Jiangsu Academy of Agricultural Sciences and was self-pollinated to generate F_2_ lines that were subsequently used as mapping populations. In 2009–2010, the F_2_ lines were grown at the experimental station in Lingshui, Hainan Province, and the leaves of the F_2_ lines were collected, numbered and stored at –70 °C. A total of 138 F_2_ individuals were selected and self-pollinated to generate 138 F_2:3_ families at the experimental station at Jiangsu Academy of Agricultural Sciences.

### Virus source and insect vector

Wheat plants showing typical symptoms of wheat dark-green dwarf disease^27^ were collected from Jianhu in Jiangsu Province, tested by RT-PCR^28^. The PCR-positive plants were grown in a greenhouse, which would be used in the artificial inoculation identification.

RSV-free SBPH specimens were acquired according to the method of Zhou *et al.*^27^. The larvae were fed on wheat plants infected with RBSDV for 2–3 d to acquire the virus and were then transferred to Wuyujing No. 3 seedlings (1.5-leaf stage to 2-leaf stage) and reared for 10–12 d at 25 °C so that the virus could pass through the circulative period. The rate of SBPH infection by RBSDV was detected by dot-ELISA^29^.

### SBPH resistance evaluation

To determine the resistance mechanism of Tetep, antibiosis and non-preference tests were performed according to standard evaluation methods for this rice variety under greenhouse conditions^10^.

### RBSDV resistance evaluation

The two parents were evaluated to determine their resistance against RBSDV using the field test and the artificial inoculation test. In total, 138 F_2:3_ families were evaluated to determine their resistance against RBSDV using the artificial inoculation test.

The experimental farms of JH and GY in Jiangsu Province were selected as disease nurseries for the field test. In 2010, the seeds of the two parents were sown, and the seedling plot was encircled by wheat fields where SBPH lived on wheat plants throughout the winter. The SBPH individuals moved from the wheat fields into the neighboring fields when the wheat hosts were harvested. At the peak stage of immigration of the first generation, 500 SBPH individuals were collected from the disease nurseries at random, 100 were randomly selected, and the rate of SBPH infection by RBSDV was detected by dot-ELISA^29^. No pesticides or antivirals were sprayed during the experiment. Seedlings that were approximately 30 days old were then transplanted into the experimental plot, and the landraces were arranged into single-row plots. Individuals with the typical symptoms of rice black-streaked dwarf disease were considered as susceptible plants^1^, whereas those without symptoms of rice black-streaked dwarf disease were considered to be resistant plants. Resistance against RBSDV was evaluated based on the incidence of disease (the number of RBSDV-infected plants/the total number of plants counted ×100). A survey of the incidence was conducted during the first epidemic period of RBSDV, and the second survey was conducted after 7 d. The data were processed using DPS 7.05 software.

In the artificial inoculation test, 30 seeds of each parent and the F_2:3_ lines were sown in 1000 mL beakers in the growth room of Jiangsu Academy of Agricultural Sciences with between 35 to 45 percent humidity and temperatures between 25 to 30 degrees C. At approximately the 1.5-leaf stage, 25 healthy seedlings were inoculated for 48 h by inoculating 4 SBPH per seedling. SBPH individuals were manually removed after 12 h to ensure uniformity in the inoculation intensity^14^. The seedlings were transplanted into an experimental area consisting of cement pools at Jiangsu Academy of Agricultural Sciences, which were managed under our standard practices without pesticide or antiviral spraying during the rice-growing period. The survey and data processing methods were same as those used for the field test.

### Genotyping, linkage map construction and QTL analysis

Total DNA was extracted from the leaves of the two parents, the F_1_ generation and 138 F_2_ lines using the CTAB method described by Murray *et al.*^30^. SSR markers (842 pairs) obtained from Gramene (http://www.gramene.org), and markers that were polymorphic between the two parents were tested. PCR amplification was performed as described by Septiningsih *et al.*^31^, and the PCR products were detected using 8% denaturing polyacrylamide gel electrophoresis. The polymorphic markers were used for genotype analysis of the F_2_ lines, and a linkage map was assembled by employing MAPMAKER/EXP 3.0 software^32^,^33^. Marker distances in centimorgans (cM) were calculated using the Kosambi function^34^.

Composite interval mapping (CIM) was applied to analyze the phenotypic and genotypic data, and the QTLs responsible for resistance to RBSDV were detected using Windows QTL Cartographer V2.5 software^35^. Experiment-wide significance (P < 0.05) threshold values of the LOD scores were determined with 1000 permutations for putative QTL detection. The threshold LOD score was 2.0 at a significance level of P = 0.05 for CIM. Additive effects and explanations of the phenotypic variance for each QTL were also obtained using this software.

## Additional Information

**How to cite this article**: Zhou, T. *et al.* Genetic analysis and molecular mapping of QTLs for resistance to rice black-streaked dwarf disease in rice. *Sci. Rep.* **5**, 10509; doi: 10.1038/srep10509 (2015).

## Figures and Tables

**Table 1:** Resistance different rice varieties to SBPH.

**Variety**	**Antibiosis**	**Non-preference**
Wuyujing No. 3	95.00 ± 0.020A	4.16 ± 0.943^a^
Huaidao No. 5	93.75 ± 0.066A	3.93 ± 0.617^ab^
Tetep	87.50 ± 0.032A	2.59 ± 0.320^bc^
IR50	68.75 ± 0.024B	1.23 ± 0.039^c^

1) Non-preference was indicated by the total number of SBPH individuals that had settled on a plant for three days.

2) Antibiosis was indicated by the average SBPH survival rate at five days after introduction.

3) Means with the same capital letter are not significant at 0.01 by DMRT.

Means with the same lowercase letter are not significant at 0.05 by DMRT.

**Table 2:** Parameters associated with QTLs for RBSDV resistance identified in the F_2_ population.

**QTL**	**Chr.**	**Position cM**	**Marker interval**	**LOD**	**Percentage of the variance explained**	**Additive**	**Loci reported**^1^	**Ref.**[2]
*qRBSDV-3*	3	19.1	RM5626-RM7097	4.07	17.5%	12.57	*qRBSDV3b*	a
							*qSBPH3-a*	b
							*Qsbph3d*	c
*qRBSDV-10*	10	8.8	RM216-RM311	2.24	0.3%	–0.02	*qSTV-10*	d
*qRBSDV-11*	11	21.8	RM202-RM7120	2.21	12.4%	9.75	*qStv11*	e
							*qSTV11-f*	f

^1^QTL or gene reported to confer resistance to RBSDV, RSV and SBPH.

^2^The letters a, b, c, d, e and f represent the reports by Zheng *et al.*^13^, Sun *et al.*^18^, Duan *et al.*^19^, Sun^20^, Ding *et al.*^22^, and Sun^20^, respectively.
